# D-Arg^0^-Bradykinin-Arg-Arg, a Latent Vasoactive Bradykinin B_2_ Receptor Agonist Metabolically Activated by Carboxypeptidases

**DOI:** 10.3389/fphar.2018.00273

**Published:** 2018-03-27

**Authors:** Hélène Bachelard, Xavier Charest-Morin, François Marceau

**Affiliations:** ^1^Division of Endocrinology and Nephrology, Centre Hospitalier Universitaire de Québec Research Center-CHUL, Laval University, Quebec, QC, Canada; ^2^Division of Infectious Diseases and Immunity, Centre Hospitalier Universitaire de Québec Research Center-CHUL, Laval University, Quebec, QC, Canada

**Keywords:** bradykinin, angiotensin converting enzyme, arginine carboxypeptidases, B_2_ receptors, hypotension, D-Arg^0^-BK-Arg-Arg

## Abstract

We previously reported hypotensive and vasodilator effects from C-terminally extended bradykinin (BK) sequences that behave as B_2_ receptor (B_2_R) agonists activated by vascular or plasma peptidases. D-Arg^0^-BK-Arg-Arg (r-BK-RR) is a novel prodrug peptide hypothetically activated by two catalytic cycles of Arg-carboxypeptidases (CPs) to release the direct agonist D-Arg^0^-BK. N-terminally extending the BK sequence with D-Arg^0^ in the latter peptide was meant to block the second kinin inactivation pathway in importance, aminopeptidase P. The affinity of r-BK and r-BK-RR for recombinant B_2_R was assessed using a [^3^H]BK binding displacement assay. Their pharmacology was evaluated in human isolated umbilical vein, a contractile bioassay for the B_2_R, in a morphological assay involving the endocytosis of B_2_R-green fusion protein (GFP) and in anesthetized rats instrumented to record hemodynamic responses to bolus intravenous injection of both peptides. r-BK exhibited an affinity equal to that of BK for the rat B_2_R, while r-BK-RR was 61-fold less potent. In the vein and the B_2_R-GFP internalization assay, r-BK was a direct agonist unaffected by the blockade of angiotensin converting enzyme (ACE) with enalaprilat, or Arg-CPs with Plummer’s inhibitor. However, the *in vitro* effects of r-BK-RR were reduced by these inhibitors, more so by enalaprilat. In anesthetized rats, r-BK and r-BK-RR were equipotent hypotensive agents and their effects were inhibited by icatibant (a B_2_R antagonist). The hypotensive effects of r-BK were potentiated by enalaprilat, but not influenced by the Arg-CPs inhibitor, which is consistent with a minor role of Arg-CPs in the metabolism of r-BK. However, in rats pretreated with both enalaprilat and Plummer’s inhibitor, the hypotensive responses and the duration of the hypotensive episode to r-BK were significantly potentiated. The hypotensive responses to r-BK-RR were not affected by enalaprilat, but were reduced by pre-treatment with the Arg-CPs inhibitor alone or combined with enalaprilat. Therefore, *in vivo*, Arg-CPs activity is dominant over ACE to regenerate the B_2_R agonist r-BK from r-BK-RR, a prodrug activator of the B_2_R. A B_2_R agonist activated only at the level of the microcirculation by resident peptidases could be developed as an intravenously infused drug for ischemic diseases.

## Introduction

Bradykinin (BK), a short-lived peptide, is the prototype of a family of peptides, the kinins, formed by the action of kallikreins on blood kininogens; the multi-molecular kallikrein-kinin system includes 2 G-protein coupled receptors termed B_1_ and B_2_ receptors [B_1_R, B_2_R; (Leeb-Lundberg et al., 2005)]. While the B_1_R appears to have limited distribution and is generally absent in normal tissues, but is strongly induced under conditions of inflammation and tissue damage (Bouthillier et al., 1987; Marceau et al., 1988; Marceau and Regoli, 2004), the B_2_R is constitutively expressed in a variety of tissues, and account for most of the vascular and metabolic actions of BK (Regoli and Barabe, 1980; Heitsch, 2003; Veeravalli and Akula, 2004; Marketou et al., 2010; Potier et al., 2013). BK exerts a variety of actions implicated in several physiological and pathological process, such as inflammatory reactions, due to its ability to cause vasodilation, hyperemia, vascular leakage, and pain sensation (Leeb-Lundberg et al., 2005; Moreau et al., 2005). However, apart from being a pro-inflammatory mediator, BK is also recognized as a regulator of blood pressure and several vascular and renal functions, mainly triggered by the synthesis and release of the vasorelaxant, anti-hypertrophic and anti-atherosclerotic endothelial mediators nitric oxide, prostaglandins, and tissue-type plasminogen activator (Brown et al., 2000; Pretorius et al., 2003; Griol-Charhbili et al., 2005; Leeb-Lundberg et al., 2005; Moreau et al., 2005; Kakoki et al., 2007). These cardioprotective effects of BK during hypertension and other clinical and experimental conditions, such as cardiac failure, ischemia, myocardial infarction, and pulmonary hypertension are believed to be B_2_R-mediated (Heitsch, 2003; Veeravalli and Akula, 2004; Marketou et al., 2010; Sharma and Al-Banoon, 2012; Potier et al., 2013). Consequently, B_2_R agonists may have important clinical value in the treatment and prevention of various cardiovascular disorders by mimicking the beneficial effects of BK.

Inspired by a “prodrug” strategy where a therapeutic B_2_R agonist would be activated only at the level of the microcirculation by resident peptidases, we have previously explored the design of potential peptide drugs that are latent B_2_R agonists activated by peptidases in isolated vascular systems (Gera et al., 2011; Charest-Morin et al., 2014) and *in vivo* (Jean et al., 2016). One of the most interesting, BK-Arg, massively lost affinity for recombinant B_2_Rs but could regenerate active BK after reaction with arginine-carboxypeptidases (Arg-CPs) present in vascular tissue and blood plasma (Charest-Morin et al., 2014; Jean et al., 2016). The model was supported by the inhibition of BK-Arg biological activities by Plummer’s inhibitor, a high affinity blocker of Arg-CPs that is an arginine analog (Plummer and Ryan, 1981). Other BK sequences C-terminally extended with 2 residues were tested as angiotensin converting enzyme (ACE) substrates (Charest-Morin et al., 2014), but the cleavage rule(s) that lead to BK regeneration were not clear *in vivo*, possibly involving multiple peptidases (Jean et al., 2016). On the other hand, B_2_R agonists that are resistant to multiple inactivation pathways, such as the peptide B-9972 (Jean et al., 2016) or the amphibian BK homolog maximakinin (Charest-Morin et al., 2017), may not be desirable vasodilators due to their propensity to activate extravascular B_2_Rs on afferent nerve terminals, epithelia and other tissues, and are not remarkably more longer acting than BK *in vivo* when administered as intravenous boluses. BK, itself, is highly susceptible to intravascular inactivation mainly by ACE (Cyr et al., 2001; Fryer et al., 2008).

**FIGURE 1:**
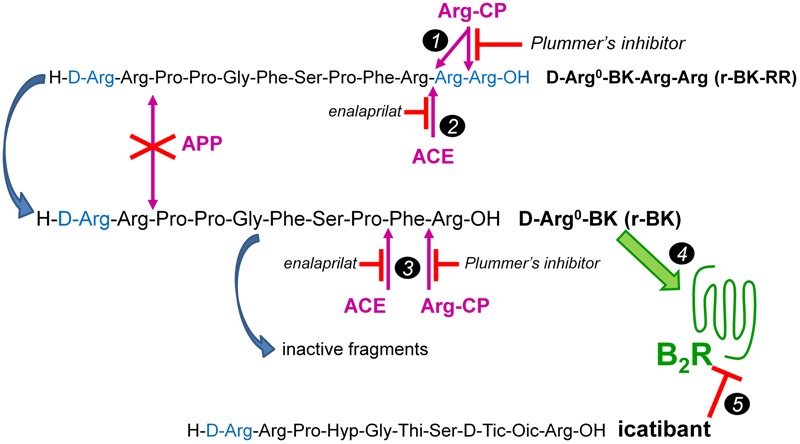
The C-terminally extended r-BK-RR sequence as potential “prodrug” agonist of the B_2_R activated by peptidases. r-BK is itself potentially degraded by several peptidases that terminate its signaling at B_2_Rs, but not by the major kininase aminopeptidase P. ACE, angiotensin converting enzyme; APP, aminopeptidase P; Arg-CPs, arginine carboxypeptidases. Marker *1*: two cycles of Arg-CPs may regenerate r-BK from r-BK-RR. Marker *2*: alternatively, ACE acting as a carboxydipeptidase may directly activate r-BK-RR. Marker *3*: in turn, Arg-CPs and ACE will limit the half-life of r-BK by attacking his unprotected C-terminus. Marker *4*: r-BK is postulated to be the only efficient high affinity B_2_R agonist in the system. Marker *5*: effects mediated at the B_2_R are abated by the BK-sequence related B_2_R antagonist icatibant. Modified from Jean et al. (2016).

We report here a second round of the development of BK prodrug/soft drug design based on a peptide that is also C-terminally extended. The basic assumption is that prolonged BK sequences massively lose affinity for the B_2_R, and also regenerate the C-terminal sequence of BK upon cleavage. Novel aspects include the block of the second kinin inactivation pathway in importance, aminopeptidase P (Cyr et al., 2001; Fryer et al., 2008), by N-terminally extending the BK sequence with D-Arg^0^; this extension is found notably in the antagonist icatibant (**Figure 1**). Thus, we have explored the possibility of a controlled release of the direct agonist D-Arg^0^-BK (r-BK) by 2 cycles of hydrolysis by Arg-CPs from D-Arg^0^-BK-Arg-Arg (r-BK-RR) (**Figure 1**). Circulating carboxypeptidase N and membrane-bound carboxypeptidase M are Arg-CPs strategically located to limit the regeneration of r-BK in the vasculature.

Although the protective role of kinins in the circulation is increasingly recognized, there have been very few attempts to use BK or a derivative in cardiovascular therapeutics. Therefore, development of a new drug class, designed to exploit vascular and blood plasma peptidases to stimulate the most desirable effects of endothelial B_2_Rs and where circulatory benefits are generated, might find application in intensive care situations where an intravenous line is available (unstable angina, myocardial infarction, perhaps decompensated congestive heart failure) and possibly, in more chronic ailments (e.g., pulmonary hypertension).

## Materials and Methods

### Drugs

Bradykinin was purchased from Bachem (Torrance, CA, United States), the B_2_R antagonist icatibant, from Phoenix Pharmaceuticals (Burlingame, CA, United States), enalaprilat dihydrate, from Kemprotec Ltd. (Maltby, Middlesbrough, United Kingdom) and Plummer’s inhibitor (mercaptomethyl- 3-guanidinoethylthiopropanoic acid) from Calbiochem (La Jolla, CA, United States; sequence of BK-related peptides in **Figure 1**). B-9972 {D-Arg-[Hyp^3^, Igl^5^, Oic^7^, Igl^8^]-BK, (Bawolak et al., 2007; Jean et al., 2016)} was a gift from Dr. Lajos Gera (University of Colorado Denver).

### Design of Novel Prodrugs

D-Arg^0^-BK (r-BK) and D-Arg^0^-BK-Arg-Arg (r-BK-RR) were custom synthesized by CanPeptide, Inc. (Pointe-Claire, QC, Canada) via standard solid-phase methodology and provided as ≥98.3% pure reagents (mass spectroscopy and HPLC analyses). The first peptide, r-BK, putatively is an N-terminally protected direct agonist of the BK B_2_R by virtue of its intact C-terminal BK sequence (**Figure 1**). The other C-terminally prolonged peptide theoretically retains little affinity for the B_2_R, which has been experimentally verified. r-BK-RR was designed as a potential prodrug needing 2 cycles of reaction with Arg-CPs to release r-BK.

### [^3^H]BK Binding Competition Assays

Affinity for the B_2_R was evaluated using a radioligand binding competition assay performed at 0°C in the presence of peptidase inhibitors that included captopril and PMSF (Charest-Morin et al., 2014). Briefly, the binding of 3 nM [^3^H]bradykinin (Perkin Elmer Life Sciences; 90 Ci/mmol) to adherent intact Human Embryonic Kidney (HEK 293) cells stably expressing the myc-tagged rat B_2_R construction (Charest-Morin et al., 2017) was applied to construct binding competition curves for a series of unlabeled peptides.

### Competition of an ACE Substrate by Synthetic Kinins

We applied a previously described enzymatic assay based on the internally quenched fluorogenic substrate Abz-Phe-Arg-Lys(Dnp)-Pro-OH, obtained from Bachem (Torrance, CA, United States), knowing that it has an approximately equal low micromolar affinity for the two separate catalytic sites of ACE (Araujo et al., 2000), to determine the affinity of the novel extended BK sequences for this peptidase. The source of enzyme was whole HEK 293a cells grown in 24-well plates and transfected as described with a vector coding for the human ACE-mCherry fusion protein, an active enzyme (Charest-Morin and Marceau, 2016). The culture medium was removed from cell wells, which were rinsed twice with PBS. The fluorogenic substrate (final concentration 20 μM), and, optionally, a competitor (BK, r-BK, or r-BK-RR in variable concentrations, or the ACE inhibitor enalaprilat 1 μM) were added in 250 μl PBS in each well; the plates were then incubated for 15 min at 37°C. At this point, the well supernatants were transferred in Eppendorf tubes, centrifuged 30 s at 12500 rpm to remove debris and 100 μl of this fluid transferred in wells of 96-well plate (black background) to read the fluorescence (excitation 320 nm, emission 420 nm) using a TECAN Infinite^®^ 200 PRO microplate reader. Results were expressed as the percent of the fluorescence in wells containing ACE-mCherry and the substrate without competitors. Controls included mock-transfected cells and enalaprilat-treated cells.

### Human Umbilical Vein Contractility Assay

The institutional research ethics board (CHU de Québec) approved the anonymous use of human umbilical cord segments obtained after elective cesarean section deliveries (file number: 2012-323). Informed written consent was obtained from mothers. Umbilical vein rings, used as a contractile bioassay for the BK B_2_R, were prepared and suspended in organ baths and submitted to equilibration in Krebs’ solution as described (Marceau et al., 1994; Gera et al., 2011). The vascular preparation was used to assess the effect of the peptidase inhibitors (introduced 30 min before the agonist) on the apparent potency of r-BK and r-BK-RR. The full cumulative concentration-effect curves were recorded for each peptide; a large concentration of BK (9.4 μM) was added to record the maximal contractile effect mediated by the B_2_Rs for low potency agonists. Tissues were used only once and discarded; controls curves were obtained from other vascular rings from the same vein.

### Microscopy of B_2_R-GFP

Epifluorescence of GFP-tagged rabbit B_2_R (B_2_R-GFP) was observed in HEK 293 cells that stably express this construction, in order to detect the effect of r-BK and of r-BK-RR on receptor endocytosis and recycling, a morphological response to receptor stimulation. This system was previously shown to exhibit BK-induced endosomal internalization of the fluorescent receptor, maximal 30 min after stimulation but with gradual recycling to the cell surface in 1–3 h (Bachvarov et al., 2001; Charest-Morin et al., 2013). This system, based on a serum-containing culture medium, contains ACE and Arg-carboxypeptidase activities (Bachvarov et al., 2001; Charest-Morin et al., 2014). r-BK or r-BK-RR were added to 35-mm Petri dishes containing the cells with optional pre-treatment with peptidase inhibitors and the cells were observed as described after 30 min of incubation at 37°C (Charest-Morin et al., 2014). The cells were observed in epifluorescence microscopy at 1000× magnification and photographed using an Olympus BX51 microscope coupled to a CoolSnap HQ digital camera (filters for GFP and fluorescein: excitation 460–500 nm, emission 510–560 nm, objective lens 100× oil UPlanApo, Olympus).

### *In Vivo* Hemodynamics in Anesthetized Rats

All surgical and experimental procedures were reviewed and approved by the Animal Care and Handling Committee of Laval University, in accordance with the Canadian Council on Animal Care. Experiments were performed on male Sprague-Dawley rats (300–375 g) purchased from Charles River Laboratories (St-Constant, QC, Canada). The rats were housed in a light-controlled (12:12-h light-dark cycle (lights on at 0600)) and temperature-regulated room (22 ± 1°C). Animals had free access to normal chow diet and tap water. They were allowed to acclimate to their environmental conditions for 1 week prior to being studied. At the end of the acclimation period, the rats were anesthetised with sodium pentobarbital (50 mg kg^-1^, i.p., supplemented as required) and had one catheter implanted into the right jugular vein [for intravenous (i.v.) injection] and one into the left femoral artery [for direct and continuous measurement of blood pressure and heart rate as previously described (Jean et al., 2016)]. Experiments started at least 20 min following the end of surgery in anesthetised rats.

Baseline measurements of heart rate and phasic and mean arterial blood pressure were recorded over a period of 15 min in anesthetized rats. A dose response curve was then obtained by recording changes in blood pressure and heart rate elicited by i.v. injection of peptide vehicle followed by increasing doses (0.025–12.8 μg/kg) of r-BK or r-BK-RR. Peptides were dissolved in isotonic saline (0.9% NaCl) containing 0.1% BSA to prevent the adsorption of peptide to the glassware and plastic surfaces. All i.v. injections were given as 100 μl boluses which were washed in with a further 100 μl of saline (the dead space of the catheter). Only one peptide was tested per group of rats and each injection started with saline-BSA 0.1% followed by the lowest dose of peptide. The next dose was administered once all recorded cardiovascular parameters had returned to baseline after the previous injection (usually 2–10 min). At the end of the experiments each animal was euthanized with an overdose of sodium pentobarbital (240 mg/kg, i.v.).

The mechanism subserving the cardiovascular responses to random i.v. injections of r-BK and r-BK-RR at the dose of 1.6 μg/kg, was evaluated in rats pretreated with specific antagonists. In these experiments, the rats were separated in four groups depending on the antagonist tested, and the cardiovascular responses to the peptides agonists were compared with those elicited in the untreated control group of rats. In the first group of treated rats, the long acting and selective B_2_R antagonist, icatibant (Hoe 140) (D-Arg-[Hyp^3^, Thi^5^, D-Tic^7^, Oic^8^]) bradykinin (Hock et al., 1991; Wirth et al., 1991; Rhaleb et al., 1992; Marceau et al., 1994) was intravenously administered as bolus (75 μg/kg, 0.1 ml) following a 10 min period of baseline measurements of heart rate and blood pressure. Fifteen minutes later, recording changes in blood pressure and heart rate elicited by i.v. injection of peptide vehicle (saline-BSA 0.1%) followed by random injections of r-BK and r-BK-RR, were made as described above. The next peptide was administered once all recorded cardiovascular parameters had returned to baseline after the previous injection. Further experiments were made in a second group of rats pretreated with the ACE inhibitor, enalaprilat. In these experiments, enalaprilat was intravenously administered as bolus (0.1 m/kg, 0.1 ml) 15 min before the i.v. injections of peptide vehicle and both customized peptides, as above. Further experiments were also carried out in rats pretreated with the Plummer’s inhibitor (mercaptomethyl-3-guanidinoethylthiopropanoic acid) a high affinity inhibitor of arginine carboxypeptidases that is an arginine analog (Plummer and Ryan, 1981). The inhibitor was intravenously administered as bolus (0.75 mg/kg, 0.1 ml) followed 15 min later by the i.v. injections of saline-BSA 0.1%, and then r-BK and -r-BK-RR (1.6 μg/kg) in random order. A fourth group of rats was pretreated with a combined i.v. injection of enalaprilat (0.1 mg/kg) and the Plummer’s inhibitor (0.75 mg/kg) 15 min before the randomized i.v. injections of both customized peptides, as above. The doses of different inhibitors were based on preliminary experiments and from previous studies performed by us and others (Ishida et al., 1989; Wirth et al., 1991; Muto et al., 2003; Jean et al., 2016). At the end of the experiments the rats were euthanized with an overdose of sodium pentobarbital (240 mg/kg, i.v.).

### Data Analysis

Results are presented as means ± SEM. Radioligand binding data were fitted by non-linear regression to a one-site competition equation using a least-square method (Prism 5.0, GraphPad Software Inc., San Diego, CA, United States) and IC_50_ values calculated from this procedure. The same computer program was used to draw concentration-effect curves generated with the contractility assay (least square fitting of sigmoidal dose-response equation with variable slope) and to derive contractile EC_50_ values. Data describing the dose-response relationship of novel peptides vs. hypotension and heart rate changes, the baseline values of heart rate and mean arterial blood pressure and the effect of drugs on the hemodynamic responses to r-B K and r-BK-RR were assessed by using one-way analysis of variance (ANOVA) followed by the Dunnett’s test (repeated comparison with a common control; Prism 5.0 software).

## Results

### Affinity of Peptides of the r-BK Series for the Rat B_2_R

**FIGURE 2:**
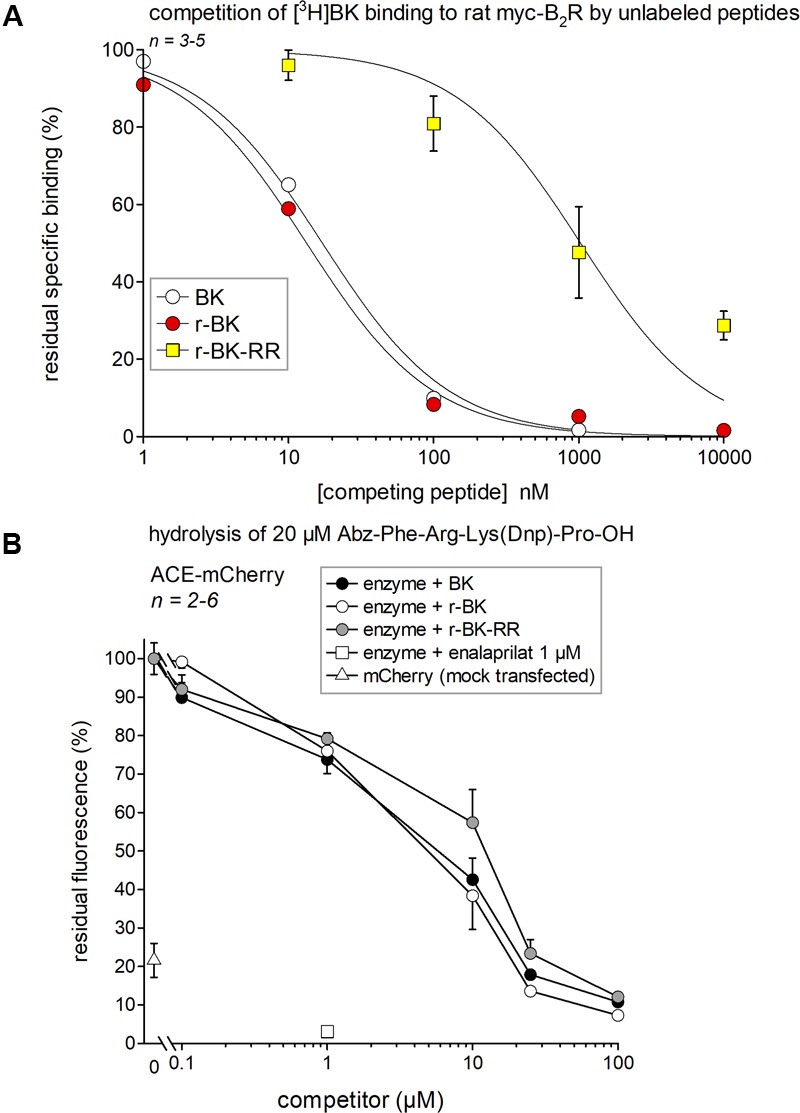
Affinity estimates of the novel peptides of the r-BK series and of BK for molecular targets. **(A)** Affinity for rat recombinant myc-B_2_R as determined by competition of [^3^H]BK binding. IC_50_ values and their 95% confidence limits are reported in **Table 1**. **(B)** Competition of a fluorogenic substrate of recombinant ACE-mCherry by the kinins. Controls included cells without ACE (mock-transfected with mCherry) and cells treated with the ACE inhibitor enalaprilat, as indicated. Results are expressed as the mean ± SEM of 2–6 replicates.

A [^3^H]BK binding competition assay performed at water-ice temperature in the presence of protease/peptidase inhibitors was exploited to determine the true receptor affinity of r-BK and r-BK-RR. Thus, r-BK was found to exhibit an affinity practically equal to that of BK for the rat myc-B_2_R construction (**Figure 2A**; IC_50_ values and their 95% confidence limits in **Table 1**). As expected, the C-terminal prolongation of r-BK decreased the receptor affinity, 61-fold for r-BK-RR (**Figure 2A** and **Table 1**). Thus, any significant activity of the C-terminally prolonged analog in a B_2_R-mediated bioassay of BK must be dependent of the regeneration of the direct agonist r-BK by precise cleavage rules.

**Table 1:** Nanomolar IC_50_ values and their 95% confidence limits (between parentheses) derived from the competition assay for [^3^H]BK binding to B_2_Rs (**Figure 2A**).

Peptide	[^3^H]BK binding competition, rat myc-B_2_R
BK	17.2 (13.6–21.6)
r-BK	13.3 (10.3–17.3)
r-BK-RR	1048 (448–2455)

### Affinity of Peptides of the r-BK Series for the Recombinant ACE

If r-BK or r-BK-RR were ACE substrates, it would be predicted that they would compete for the hydrolysis of another ACE substrate. Thus, we used the fluorogenic ACE substrate Abz-Phe-Arg-Lys(Dnp)-Pro-OH and recombinant ACE-mCherry to determine, by competition, the affinity of r-BK and r-BK-RR over that of BK for ACE. As shown in **Figure 2B**, BK and r-BK competed with a similar affinity for the recombinant enzyme in a concentration-dependent manner, exhibiting nearly complete inhibition at a concentration equal to that of the substrate (20 μM). Unexpectedly, r-BK-RR was also active in this assay, only slightly less potent than the two other peptides, indicating that r-BK-RR may be an ACE substrate. Controls made with cells without ACE (mock-transfected with mCherry) catalyzed the reaction to a small extent that may represent a contamination of cells by ACE from the serum-containing culture medium. ACE-expressing cells treated with enalaprilat did not catalyze the reaction.

### Pharmacological Profile of Peptides of the r-BK Series in an Isolated Vascular Preparation

**FIGURE 3:**
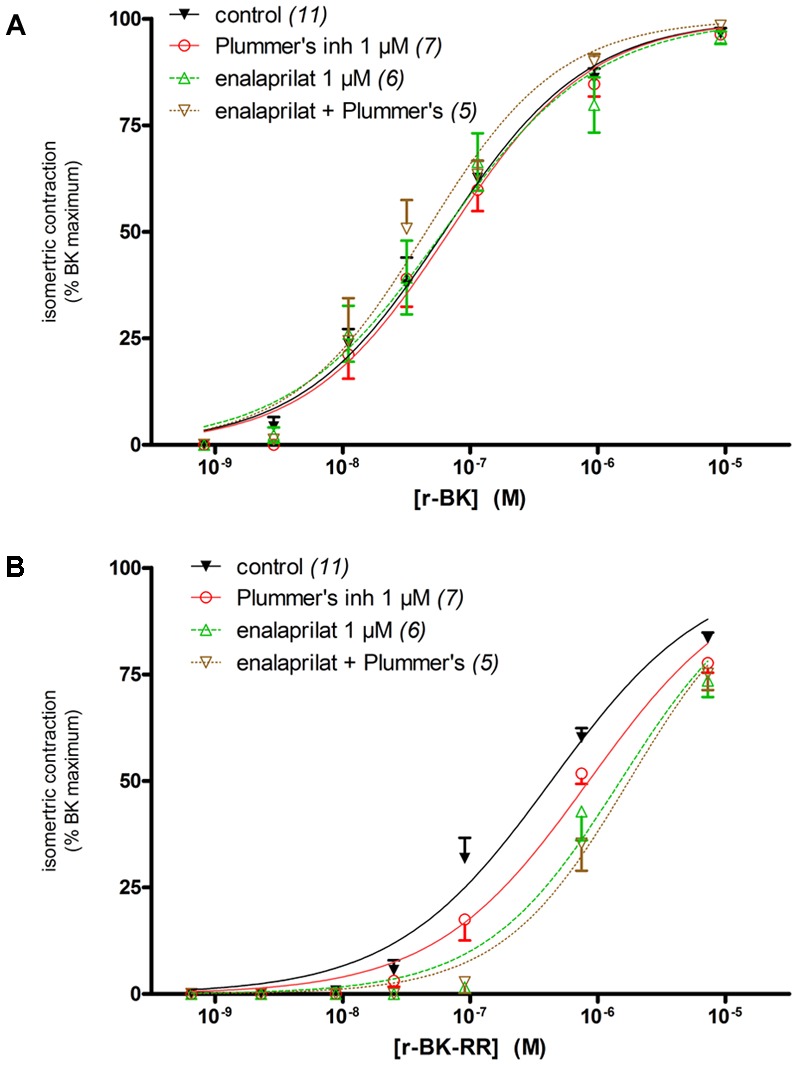
Contractility studies in the human isolated umbilical vein, a bioassay for the B_2_R, for r-BK and r-BK-RR. **(A)** Cumulative concentration-effect curve for r-BK as modified by peptidase inhibitors. **(B)** Cumulative concentration-effect curve for r-BK-RR as modified by the indicated peptidase inhibitors. Separate tissues from the same individuals were used as controls. Values are means ± SE of the number of replicates indicated between parentheses. The contractile EC_50_ values and their 95% confidence limits are reported in **Table 2**.

The human isolated umbilical vein is a contractile bioassay for the endogenous B_2_R, and was exploited to further study the pharmacology of r-BK and R-BK-RR. Cumulative concentration-effect curves were constructed for r-BK and its homolog r-BK-RR based on the contractility of this preparation (**Figures 3A,B**; IC_50_ values and their 95% confidence limits reported in **Table 2**). Specific peptidase inhibitors were used in separate tissues, as indicated. r-BK was a potent contractile agent essentially unaffected by the blockade of ACE with enalaprilat, and/or Arg-CPs with Plummer’s inhibitors. On a molar basis, r-BK-RR was about sevenfold less potent than r-BK. This peptide was designed to regenerate r-BK after 2 cycles of reaction with Arg-CPs (**Figure 1**), but the corresponding inhibitor, Plummer’s inhibitor, only slightly decreased the apparent potency of r-BK-RR (**Figure 3B** and **Table 2**). Unexpectedly, the ACE inhibitor enalaprilat was more effective to shift the concentration-effect curve of r-BK-RR to the right by a factor 3.5; the likely explanation is the removal of the Arg-Arg extension in a single step by this carboxydipeptidase (**Figure 1**). The combination Plummer’s inhibitor + enalaprilat was not significantly more effective than enalaprilat alone (**Figure 3**) and the residual effect of r-BK-RR in the presence of ACE blockade may approach the direct micromolar affinity of the peptide for B_2_R, as suggested by its potency in the [^3^H]BK binding competition assay (**Figure 2A**). Thus, these results support a metabolic activation by ACE of the latent B_2_R agonist, r-BK-RR, in this preparation.

**Table 2:** Nanomolar EC_50_ values and their 95% confidence limits (between parentheses) derived from the human umbilical vein contractility assays (**Figure 3**).

Inhibitor	Peptide	
	r-BK	r-BK-RR
Control	62.4 (49.7–78.4)	431 (348–534)
Plummer’s inhibitor 1 μM	68.4 (52.0–90.1)	850 (683–1057)
Enalaprilat 1 μM	61.6 (41.9–90.5)	1506 (1129–2009)
Enalaprilat 1 μM + Plummer’s inhibitor 1 μM	45.1 (32.8–62.0)	1760 (1377–2250)

### Effects of r-BK and r-BK-RR on B_2_R-GFP Cycling

**FIGURE 4:**
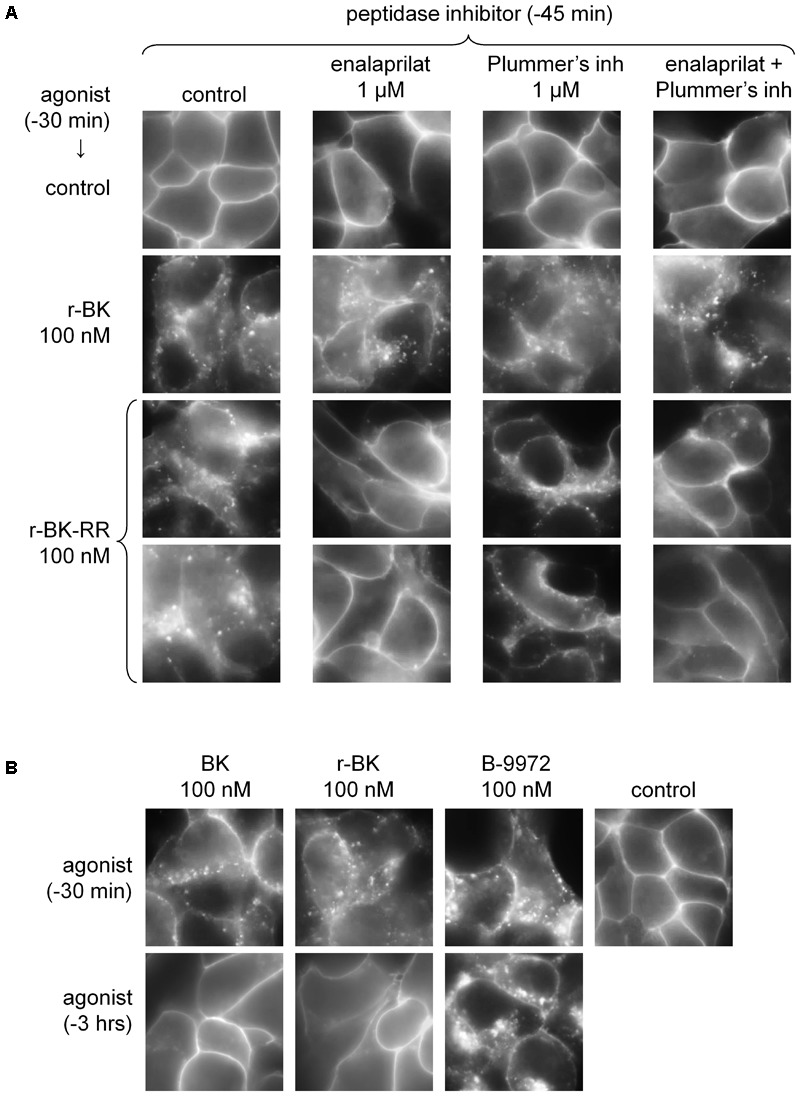
Epifluorescence microscopy studies of live HEK 293 cells stably expressing B_2_R-GFP. **(A)** Cells were stimulated for 30 min with r-BK or r-BK-RR (100 nM) with optional pretreatment with a peptidase inhibitor, as indicated. Control cells generally exhibit sharply defined plasma membrane-associated green fluorescence. Two different fields are shown for r-BK-RR. **(B)** Lack of persistence of the translocation of fluorescent receptor from the plasma membrane to endosomal structures in cells stimulated with BK or r-BK for 30 min or 3 h, as indicated. B-9972 is an inactivation-resistant agonist with a persistent morphologic response. Original magnification 1000×.

The stable transfectant HEK 293 cell line expressing B_2_R–GFP at the level of the plasma membrane was applied to detect the effect of r-BK and r-BK-RR on receptor endocytosis and recycling, a morphological response to receptor stimulation. Thus, after 30-min stimulation period with r-BK (100 nM; **Figure 4A**) in the serum-containing culture medium at 37°C, the fluorescent receptor was internalized. The cell morphology in green epifluorescence was that of disrupted plasma membrane continuity with abundant cytosolic inclusions close to the plasma membrane and discrete intracellular structures. This assay was also applied to r-BK-RR, which exerts, at 100 nM, little competition on the binding of [^3^H]BK to B_2_R: (**Figure 2A**). After 30 min of treatment with r-BK-RR, the translocation of B_2_R-GFP-associated fluorescence from the plasma membrane to endosomes was similar to that seen with r-BK, observed in the vast majority of cells (**Figure 4A**).

While HEK 293 cells do not express ACE unless transfected with the corresponding expression vector (Morissette et al., 2008), evidence was provided that their serum-supplemented culture medium contains soluble ACE (Bachvarov et al., 2001), as well as soluble Arg-CPs activity (Charest-Morin et al., 2014). The specific inhibitors of theses peptidases, enalaprilat and Plummer’s inhibitor, respectively, were applied separately or together, 15-min before an additional 30-min treatment with either peptide in additional experiments reported in **Figure 4**. While none of these inhibitors influenced the >90% proportion of cells that exhibited r-BK-induced internalization of B_2_R-GFP, treatment with enalaprilat alone or combined with Plummer’s inhibitor was found to suppress the effect of r-BK-RR (**Figure 4A**), consistent the metabolic activation of r-BK-RR by ACE. Treatment with Plummer’s inhibitor alone had no effect on r-BK-induced internalization of the fluorescent receptor, but had only a partial effect on r-BK-RR-induced translocation of plasma membrane fluorescence to endosomal structures (**Figure 4A**).

Bradykinin induces the rapid endocytosis of the B_2_R-GFP construction toward the endosomal pathway in HEK 293 cells, but this is followed by extensive recycling to the plasma membrane following 3 h of incubation (Bachvarov et al., 2001). This is replicated in experiments reported in **Figure 4B**, where BK was compared to r-BK and the inactivation-resistant B_2_R agonist B-9972. The effect of r-BK was reversible as a function of incubation time, as that of BK, while B-9972 elicits a prolonged endocytosis of B_2_R-GFP (Bawolak et al., 2007).

### *In Vivo* Hemodynamic Responses to r-BK and Its C-Terminally Extended Homolog, r-BK-RR

**FIGURE 5:**
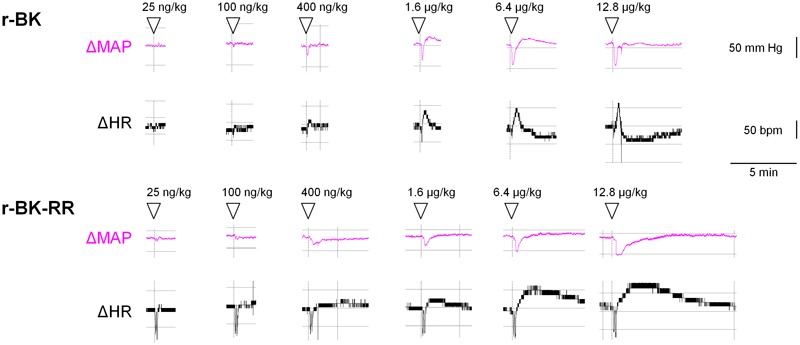
Typical tracing of the successive bolus injection effects of peptides of the r-BK series in anesthetized rats. Abscissa: time; ordinate: changes in arterial pressure (mm Hg) or heart rate (beats per minute).

**FIGURE 6:**
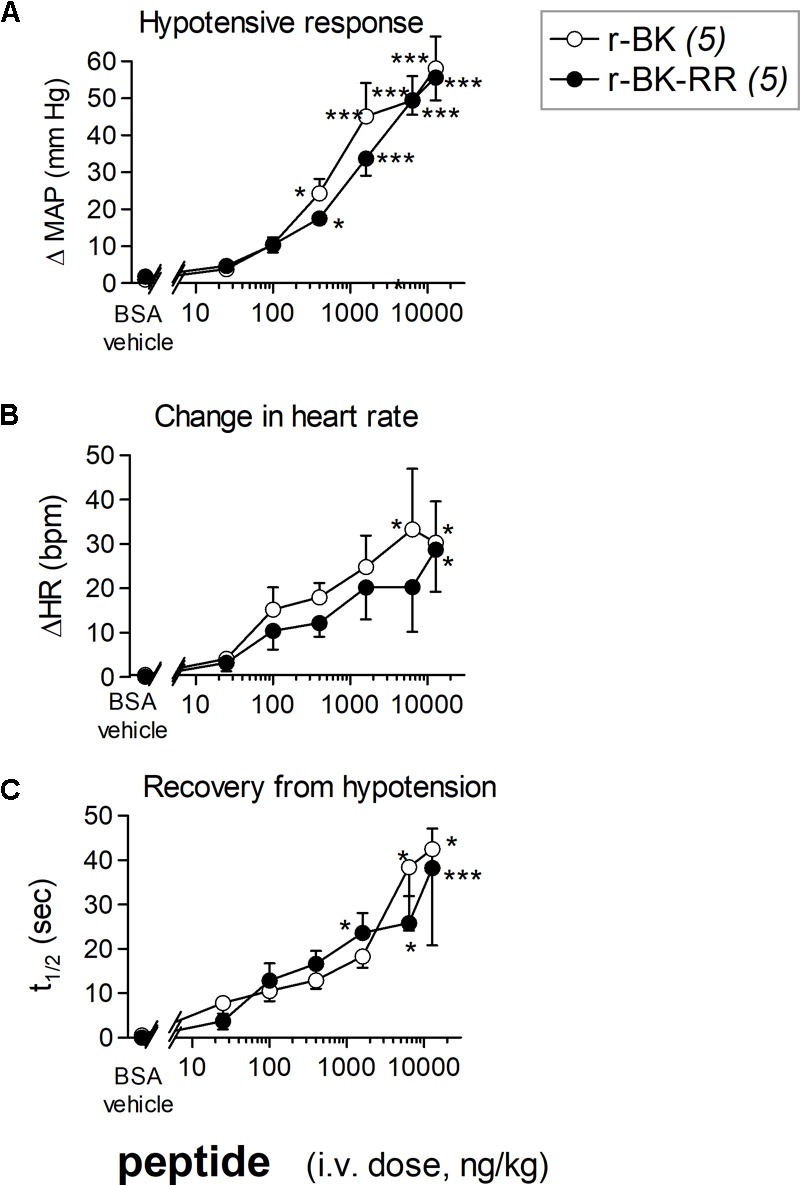
Hemodynamic responses in anesthetized rats treated with increasing doses of a peptide of the r-BK series. **(A)** Maximal hypotensive responses to i.v. bolus injections of increasing doses of r-BK and r-BK-RR. ANOVA indicated that, for each group of rats, the effects of the doses differed (*P* < 10^-4^ for each peptide). Dunnett’s test for difference from the effect of the saline-BSA vehicle: ^∗^*P* < 0.05, ^∗∗∗^*P* < 0.001. **(B)** Change in heart rate at the moment of maximal hypotensive effect. The ΔHR values related to the different doses of r-BK and r-BK-R-R were heterogenous (ANOVA, *P* < 0.05 in each case; Dunnett’s test vs. vehicle represented as in **A**). **(C)** Time to recover one half of the hypotensive effect after reaching the maximal value after bolus injection of each dose of peptides. ANOVA indicated that, for each group of rats, the effects of the doses differed (ANOVA, *P* < 0.05 and 0.001, respectively; Dunnett’s test vs. vehicle represented as in **A**).

We previously described the brief hypotensive responses associated with tachycardia in response to the i.v. injection of increasing doses of BK in anesthetized rats and the strong potentiation of the responses following pharmacologic ACE blockade (Jean et al., 2016). Using the same methods, we found that intravenous injection of increasing doses of r-BK, as bolus in anesthetized rats, produces dose-dependent hypotensive and tachycardiac responses; comparison with the effects of BK assayed under the same experimental conditions shows that r-BK is 2,5–3-fold more potent than BK as an hypotensive agent in anesthetized rats (Jean et al., 2016). In the present experiments, r-BK and r-BK-RR are virtually equipotent hypotensive agents (typical blood pressure tracings, **Figure 5**; dose-response curves, **Figure 6A**). These hypotensive responses are significantly greater than that of the BSA-saline vehicle for peptide doses greater or equal to 400 ng/kg. Further, the two peptides exhibit similar rapid action and cessation of action (half-time for the recovery of the hypotensive episodes; **Figure 6C**). After a brief bradycardic episode that follows by the bolus injection (an injection artifact), the major effect of r-BK and r-BK-RR on the heart rate usually was a brief tachycardic response, statistically significant only at the highest tested doses (**Figures 5, 6B**). This suggests a particularly effective conversion of r-BK-RR into the active peptide r-BK *in vivo*.

### Effects of Icatibant and Peptidase Inhibitors on Cardiovascular Responses to r-BK and r-BK-RR

Baseline values for mean arterial blood pressure and heart rate measured in the untreated control group or 15 min after i.v. pretreatment with icatibant or the peptidase inhibitors are shown in **Table 3**. While no significant changes in basal values of MAP were noted between the treated groups and the untreated control group, slight but significant increases in basal heart rate were noted between the treated groups and the control group.

**Table 3:** Basal cardiovascular parameters in anesthetized, pre-treated rats represented in **Figure 7**.

Pre-treatment	Mean arterial blood pressure	Heart rate	*n*
Control	93.2 ± 3.3	352 ± 10	13
Icatibant	93.7 ± 3.3	396 ± 10^∗^	10
Enalaprilat	89.8 ± 4.1	412 ± 11^∗∗^	9
Plummer’s inhibitor	90.3 ± 3.6	407 ± 10^∗∗^	8
Enalaprilat + Plummer’s inhibitor	87.8 ± 3.9	406 ± 13^∗∗^	10
ANOVA	N.S.	*P* < 0.001	

*^∗^P < 0.05, ^∗∗^P < 0.01, Dunnett’s test vs. control.*

**FIGURE 7:**
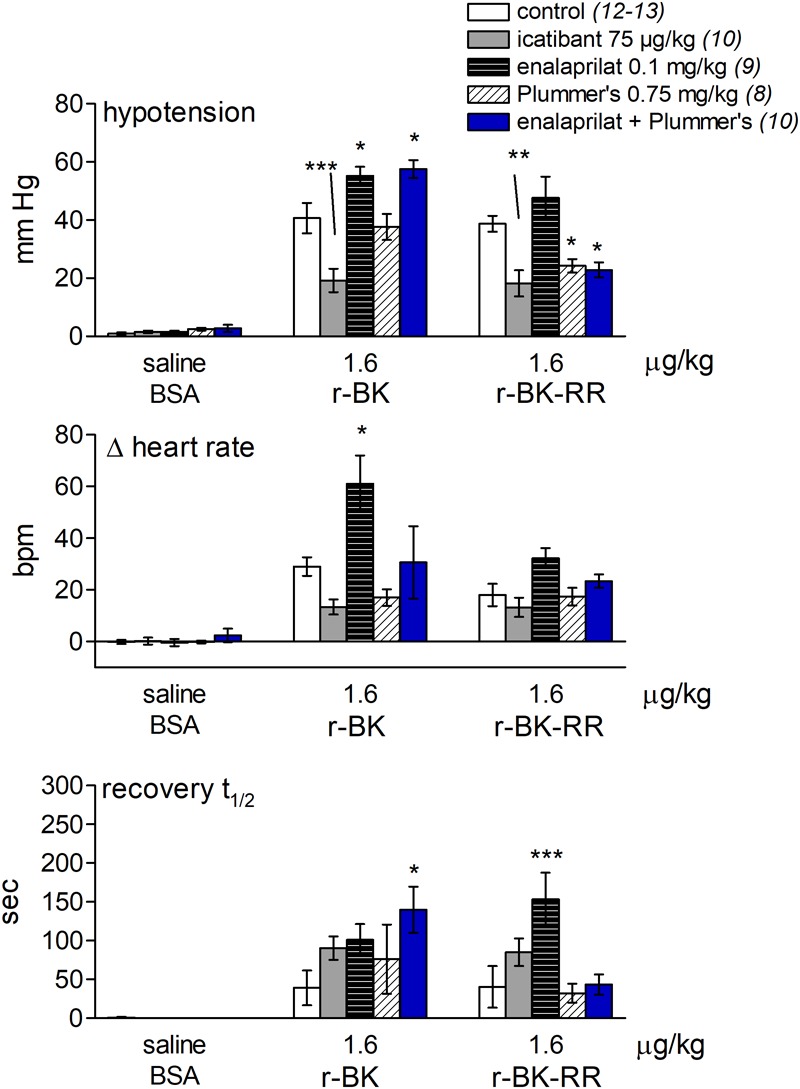
Bar graph showing the effects of icatibant and peptidases inhibitors on the cardiovascular responses to i.v. bolus injections of saline-BSA vehicle, r-BK or r-BK-RR in anesthetized rats. The **(upper)** panel represent the effect of i.v. pretreatment with icatibant (0.75 mg/kg), enalaprilat (0.1 mg/kg), Plummer’s inhibitor (0.75 mg/kg) or enalaprilat (0.1 mg/kg) + Plummer’s inhibitor (0.75 mg/kg) on the maximal hypotensive response to i.v. bolus injections of 1.6 μg/kg of r-BK and r-BK-RR in anesthetized rats. The pretreatment with each inhibitor was given 15 min before starting with the control injection of vehicle. The **(middle)** panel represent the maximal tachycardic response during hypotensive episodes induced by the indicated peptides and reported in the **(upper)** panel. The **(bottom)** panel represent the recovery half-time for hypotensive episodes induced by the indicated peptides and reported in the **(upper)** panel. In all panels, values are means ± SEM shown by vertical lines and the number of determinations in the different group of rats is given between parentheses for controls and each inhibitor. ANOVA indicated that specific sets of values differed significantly between them (*p* < 0.05): the effects of r-BK on all three readouts and those of r-BK-RR on blood pressure and recovery time. Dunnett’s test was applied within these sets of drug pretreatments vs. controls values. ^∗^*p* < 0.05, ^∗∗^*p* < 0.01, ^∗∗∗^*p* < 0.001.

**FIGURE 8:**
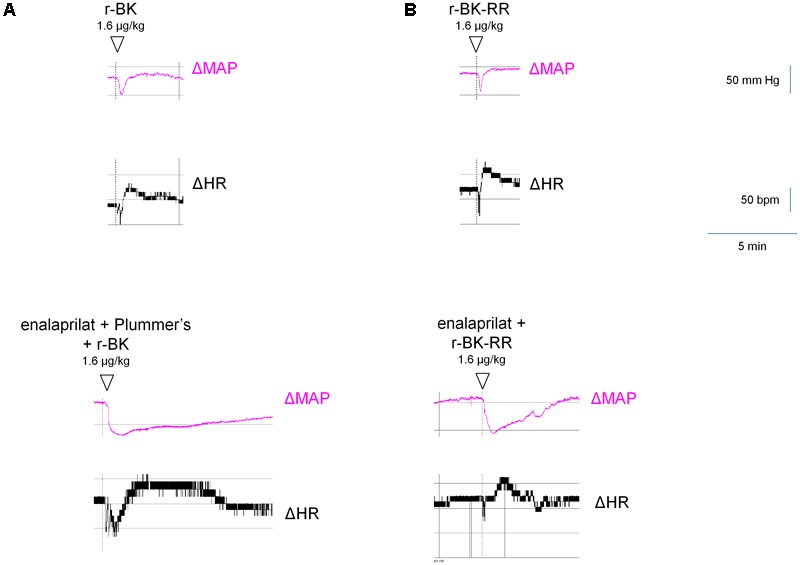
Representative traces showing the mean arterial blood pressure and heart rate responses to i.v. bolus injections of r-BK **(A)** or r-BK-RR **(B)** at the dose of 1.6 μg/kg in untreated and drug-pretreated anesthetised rats. Enalaprilat (0.1 mg/kg), with or without Plummer’s inhibitor (0.75 mg/kg) as indicated, was i.v. given as bolus 15 min before starting the injections of r-BK or r-BK-RR. Abscissa: time; ordinate: arterial pressure (mm Hg).

As shown in **Figure 7** and consistent with its direct agonist action on B_2_R, the hypotensive responses to r-BK (1.6 μg/kg) were significantly inhibited by pretreatment with icatibant (*P* < 0.001, Dunnett’s test), while the heart rate response and the duration of the residual hypotensive episode were comparable to those seen in the untreated group. In rats pretreated with enalaprilat, the hypotensive effect of r-BK was significantly potentiated (*P* < 0.05, Dunnett’s test) as was the tachycardic response (*P* < 0.05, Dunnett’s test). However, the duration of the hypotensive episode was not different from that seen in untreated rats (**Figure 7**). Pretreatment with the Plummer’s inhibitor alone had no effect on the hypotensive and tachycardic responses to r-BK, which is consistent with a minor role of Arg-CPs in the metabolism of BK (Cyr et al., 2001; Fryer et al., 2008). In rats pretreated with both enalaprilat and the Plummer’s inhibitor, the hypotensive effect of r-BK, as well as the duration of the hypotensive episode were both significantly potentiated (*P* < 0.01, Dunnett’s test), while the heart rate response was not different from that seen in untreated rats (**Figures 7, 8A**). Therefore, in the presence of enalaprilat, which contributes to amplify the hypotensive response to r-BK, the Plummer’s inhibitor reveals the involvement of Arg-CPs in cessation of the hypotensive episode.

The C-terminally extended analog r-BK-RR is predicted to be an indirect activator of the B_2_R, via its conversion to r-BK (**Figure 1**). Consistently, pretreatment with icatibant was found to strongly reduce the hypotensive response to intravenously administered (1.6 μg/kg) r-BK-RR (*P* < 0.01, Dunnett’s test), while the heart response or the duration of the residual hypotensive episode were not different from those of controls (**Figure 7**). r-BK regeneration from r-BK-RR should involve Arg-CPs, but perhaps also the carboxypeptidase ACE, based on *in vitro* results (**Figures 1–4**). Interestingly, pretreatment with enalaprilat had no significant effect on the hypotensive response to r-BK-RR, but it was found to significantly increase the duration of the hypotensive episode (*P* < 0.001, Dunnett’s test) (**Figures 7, 8B**). Pretreatment with the Plummer’s inhibitor alone or combined with enalaprilat was found to significantly reduce the hypotensive response to r-BK-RR, when compared to the untreated group (*P* < 0.05, Dunnett’s test) (**Figure 7**), while the heart rate response and the duration of the residual hypotensive episode were not different from those seen in untreated rats. Altogether, *in vivo* results indicate that Arg-CPs activity is dominant over ACE to regenerate a B_2_R agonist from r-BK-RR, but ACE inhibition potentiates the reaction product r-BK.

## Discussion

In the present study, we evaluated a “prodrug” peptide extended around the BK sequence, as a potential B_2_R agonist activated by peptidases present in the microcirculation. We explored the possibility of a controlled release of the direct agonist r-BK by two cycles of hydrolysis by Arg-CPs from r-BK-RR. Briefly, r-BK exhibited an affinity practically equal to that of BK for the rat B_2_R construction, while r-BK-RR was 61-fold less potent. In the vein and the B_2_R-GFP internalization assay, r-BK behaved as a direct agonist unaffected by the blockade of ACE with enalaprilat, or Arg-CPs with Plummer’s inhibitor. However, the *in vitro* effects of r-BK-RR were reduced by these inhibitors, more so by enalaprilat. In anesthetized rats, r-BK and r-BK-RR caused equipotent hypotensive effects that were inhibited by icatibant (a B_2_R antagonist). The hypotensive effects of r-BK were potentiated by enalaprilat, but not influenced by the Arg-CPs inhibitor, which is consistent with a minor role of Arg-CPs in the metabolism of r-BK. However, in rats pretreated with both enalaprilat and Plummer’s inhibitor, the hypotensive responses and the duration of the hypotensive episode to r-BK were significantly potentiated. The hypotensive responses to r-BK-RR were not affected by enalaprilat, but were reduced by pre-treatment with the Arg-CP inhibitor alone or combined with enalaprilat, indicating that, *in vivo*, Arg-CPs activity is likely dominant over ACE to regenerate the B_2_R agonist, r-BK, from r-BK-RR, a prodrug activator of the B_2_R.

The development of protease-activated “soft pro-drugs” might be an original and highly useful approach to deliver drugs in areas where protease expression is higher than in normal. This might contribute to reduce off-target side effects and to prolong beneficial effects by controlled and progressive regeneration of active drug. BK, a high-affinity, direct B_2_R agonist that is also an effective ACE substrate, plays an important role in the regulation of blood pressure, renal and cardiac functions, through its effects on nitric oxide, prostaglandins and tissue plasminogen (t-PA), which effects mainly arise from endothelial cell B_2_Rs activation (Brown et al., 2000; Veeravalli and Akula, 2004; Kakoki et al., 2007; Marketou et al., 2010; Potier et al., 2013). Consequently, B_2_R agonists may have important clinical value in the treatment and prevention of various cardiovascular disorders such as hypertension, ischaemic heart disease and other. Thus, using a “prodrug” strategy design and exploiting the distribution of ectopeptidases expressed in the vasculature and blood plasma, we recently provided pharmacological evidence, that interesting vascular effects can be extracted from C-terminal extended BK sequences that behave as latent peptidase-activated B_2_R agonists (Charest-Morin et al., 2014; Jean et al., 2016). In continuity with these studies, we have designed and evaluated a new “prodrug” peptide extended around the BK sequence, as a potential peptidase-activated B_2_R agonist, *in vitro* and in anesthetized rats. Novel aspects of the present study include the block of the second kinin inactivation pathway in importance, aminopeptidase P (Cyr et al., 2001; Fryer et al., 2008), by N-terminally extending the BK sequence with D-Arg^0^. This was done to explore the possibility of a controlled and progressive release of the direct agonist r-BK by two cycles of hydrolysis by Arg-CPs from r-BK-RR.

### Pharmacological Profile of BK Extended Peptides

We found that r-BK shares similar affinity than BK for the rat B_2_R, while the C-terminal prolongation of r-BK results in a severe decrease of receptor affinity, 61-fold for r-BK-RR. Therefore, any significant vasoactive effect of the latter BK analog must derive from its cleavage, leading to a more conventional receptor agonist. r-BK-RR, was designed to regenerate active r-BK after two cycles of reaction with Arg-CPs present in vascular tissue and blood plasma. Interestingly, r-BK-RR was found to be a contractile agonist of the human umbilical vein less potent than r-BK but more potent than anticipated from the radioligand binding competition assay. Moreover, the contractile potency of r-BK-RR was only slightly decreased by Plummer’s inhibitor, whereas the ACE inhibitor was more effective to shift the concentration-effect curve of r-BK-RR to the right, suggesting the removal of the C-terminal dipeptide in a single step by this carboxypeptidase. This finding is in line with the enzymatic assay applied to recombinant ACE indicating that r-BK-RR may be an ACE substrate. It is also consistent with the demonstration that r-BK-RR-induced internalization of B_2_R-GFP is selectively suppressed by enalaprilat in HEK 293 cells, a system where ACE is supplied by serum-containing culture medium (Bachvarov et al., 2001). The residual contractile effect of r-BK-RR in the presence of ACE blockade may largely depend on the direct micromolar affinity of the peptide for B_2_R, as suggested by its potency in the [^3^H]BK binding competition assay.

Although ACE presence in the umbilical vein is functionally revealed by the metabolic activation of prodrug peptides that regenerate BK, enalaprilat failed to potentiate r-BK in the human umbilical vein contractility (**Figure 3A**). The lack of effect of ACE inhibitors on the apparent potency of BK has been previously reported in this preparation (Marceau et al., 1994; Gobeil et al., 1996; Bawolak et al., 2007). Previous immunohistochemistry and immunofluorescence studies showed that the umbilical vein possesses a relatively thick media (>20 layers of smooth muscle cells), while immunoreactive ACE is limited to the single endothelial cell layer of the luminal surface of the vein (Koumbadinga et al., 2010; Gera et al., 2013). Therefore, considering the low endothelium/muscle ration found in human umbilical vein preparation, it is suggested that ACE is not abundant enough in the tissue to impair the equilibrium between BK concentrations in the bathing fluid and those at the vicinity of B_2_R that mediate the contraction of venous muscle cells (Marceau et al., 2010).

The B_2_R-GFP internalization assay also suggests that ACE is dominant over Arg-CPs to activate the pro-drug r-BK-RR, based on the effect of peptidase inhibitors (**Figure 4A**). Both in this cell culture setting and in the isolated umbilical vein model, the absolute and relative abundance of ACE and Arg-CPs may not fully represent their *in vivo* role at the level of resistance arterioles. Further, the translocation of the fluorescent receptor from the plasma membrane to endosomal structures is not more persistent (3 h of stimulation as compared to 30 min) in cells stimulated with r-BK than in those exposed to BK (**Figure 4B**). r-BK, unlike B-9972, appears to be a “soft drug” perhaps less prone to exert extravascular effects if infused in the circulation.

### Cardiovascular Effects of BK Extended Peptides in Rats

The most salient finding we made in anesthetized rats was the demonstration that r-BK and r-BK-RR are equipotent in causing rapid and transient and dose-related hypotensive responses and tachycardia following their systemic administration. While BK is a negative inotropic stimulus in the isolated rat heart (Rastaldo et al., 2001), massive vasodilation determines a reflex tachycardic response to BK injection *in vivo* (Jean et al., 2016). As expected, the hypotensive response to systemic administration of r-BK was greatly enhanced by pretreatment with enalaprilat (as was the tachycardia), extensively inhibited in the presence of a B_2_R antagonist and remained unchanged in the presence of the Plummer’s inhibitor (**Figure 7**), which is consistent with a minor role of Arg-CPs in the metabolism of r-BK. These results underscore the important role played by ACE, as the main r-BK-inactivating peptidase in the extracellular space. However, in rats pretreated with both enalaprilat and Plummer’s inhibitor, the hypotensive responses and the duration of the hypotensive episode to r-BK were significantly potentiated, revealing the involvement of Arg-CPs in cessation of the hypotensive episode when the metabolism of r-BK by ACE is inhibited.

Pharmacological evidence of B_2_R-mediated hypotensive response to r-BK-RR (shown to have very little direct affinity for B_2_R) was obtained as icatibant significantly inhibit the hypotensive effect of the peptide. However, in contrast to what we previously found in the human umbilical vein contractile assay, and despite the enzymatic assay indicating that r-BK-RR may be an ACE substrate, the cleavage rule leading to r-BK regeneration *in vivo* does not appear to follow a single catalytic step mediated by ACE. Indeed, in anesthetized rats, pretreatment with enalaprilat had no effect on the hypotensive response to r-BK-RR, but it was found to increase the duration of the hypotensive episode. Pretreatment with the Arg-CPs inhibitor alone or combined with enalaprilat was found to strongly reduce the hypotensive response to r-BK-RR. Altogether, the *in vivo* results indicate that Arg-CPs activity is dominant over ACE to regenerate a B_2_R agonist from r-BK-RR, but ACE inhibition potentiates the reaction product r-BK. Thus, the present findings indicate that the C-terminally extended analog r-BK-RR is an indirect activator of the B_2_R, via its conversion to r-BK, and support the basic postulated cleavage rule leading to r-BK regeneration following two catalytic step mediated by Arg-CPs (**Figure 1**).

r-BK-RR is presumably a pro-drug B_2_R agonist peptide activated by Arg-CPs expressed in the vicinity of vascular endothelial cells and a soft drug, because the active reaction product r-BK is itself cleared by ACE and in the endosomes. The development of such a drug, selective for the vascular system and stimulating the most desirable endothelial B_2_R effects, might prove to be very useful in specific intensive care situations where the stimulation of vascular B_2_Rs has been proposed to have therapeutic value, such as myocardial infarction and ischemic stroke.

## Author Contributions

HB and FM conceived and designed the experiments, analyzed the data, wrote the paper, prepared figures and/or tables, and reviewed the drafts of the paper. XC-M performed and designed some of the experiments and reviewed the drafts of the paper. All authors approved the version to be published.

## Conflict of Interest Statement

The authors declare that the research was conducted in the absence of any commercial or financial relationships that could be construed as a potential conflict of interest.

## References

[B1] Araujo M. C. Melo R. L. Cesari M. H. Juliano M. A. Juliano L. Carmona A. K. (2000). Peptidase specificity characterization of C- and N-terminal catalytic sites of angiotensin I-converting enzyme. *Biochemistry* 39 8519–8525. 10.1021/bi9928905 10913258

[B2] Bachvarov D. R. Houle S. Bachvarova M. Bouthillier J. Adam A. Marceau F. (2001). Bradykinin B_2_ receptor endocytosis, recycling, and down-regulation assessed using green fluorescent protein conjugates. *J. Pharmacol. Exp. Ther.* 297 19–26. 11259523

[B3] Bawolak M. T. Gera L. Morissette G. Stewart J. M. Marceau F. (2007). B-9972 (D-Arg-[Hyp3, Igl5, Oic7, Igl8]-bradykinin) is an inactivation-resistant agonist of the bradykinin B_2_ receptor derived from the peptide antagonist B-9430 (D-Arg-[Hyp3, Igl5, D-Igl7, Oic8]-bradykinin): pharmacologic profile and effective induction of receptor degradation. *J. Pharmacol. Exp. Ther.* 323 534–546. 10.1124/jpet.107.123422 17699739

[B4] Bouthillier J. Deblois D. Marceau F. (1987). Studies on the induction of pharmacological responses to des-Arg9-bradykinin in vitro and in vivo. *Br. J. Pharmacol.* 92 257–264. 10.1111/j.1476-5381.1987.tb11319.x 3676593 PMC1853641

[B5] Brown N. J. Gainer J. V. Murphey L. J. Vaughan D. E. (2000). Bradykinin stimulates tissue plasminogen activator release from human forearm vasculature through B_2_ receptor-dependent, NO synthase-independent, and cyclooxygenase-independent pathway. *Circulation* 102 2190–2196. 10.1161/01.CIR.102.18.2190 11056091

[B6] Charest-Morin X. Bachelard H. Jean M. Marceau F. (2017). Species-specific pharmacology of maximakinin, an amphibian homologue of bradykinin: putative prodrug activity at the human B_2_ receptor and peptidase resistance in rats. *PeerJ* 5:22911. 10.7717/peerj.2911 28133580 PMC5248581

[B7] Charest-Morin X. Fortin S. Lodge R. Roy C. Gera L. Gaudreault R. C. (2013). Inhibitory effects of cytoskeleton disrupting drugs and GDP-locked Rab mutants on bradykinin B_2_ receptor cycling. *Pharmacol. Res.* 71 44–52. 10.1016/j.phrs.2013.02.007 23454239

[B8] Charest-Morin X. Marceau F. (2016). Biotechnological fluorescent ligands of the bradykinin B_1_ receptor: protein ligands for a peptide receptor. *PLoS One* 11:e0148246. 10.1371/journal.pone.0148246 26844555 PMC4742067

[B9] Charest-Morin X. Roy C. Fortin E. J. Bouthillier J. Marceau F. (2014). Pharmacological evidence of bradykinin regeneration from extended sequences that behave as peptidase-activated B_2_ receptor agonists. *Front. Pharmacol.* 5:32 10.3389/fphar.2014.00032 PMC3945637 24639651

[B10] Cyr M. Lepage Y. Blais C. Jr. Gervais N. Cugno M. Rouleau J. L. (2001). Bradykinin and des-Arg(9)-bradykinin metabolic pathways and kinetics of activation of human plasma. *Am. J. Physiol. Heart Circ. Physiol.* 281 H275–H283. 11406494 10.1152/ajpheart.2001.281.1.H275

[B11] Fryer R. M. Segreti J. Banfor P. N. Widomski D. L. Backes B. J. Lin C. W. (2008). Effect of bradykinin metabolism inhibitors on evoked hypotension in rats: rank efficacy of enzymes associated with bradykinin-mediated angioedema. *Br. J. Pharmacol.* 153 947–955. 10.1038/sj.bjp.0707641 18084312 PMC2267285

[B12] Gera L. Roy C. Bawolak M. T. Bouthillier J. Adam A. Marceau F. (2011). Met-Lys-bradykinin-Ser-Ser, a peptide produced by the neutrophil from kininogen, is metabolically activated by angiotensin converting enzyme in vascular tissue. *Pharmacol. Res.* 64 528–534. 10.1016/j.phrs.2011.08.001 21864683

[B13] Gera L. Roy C. Charest-Morin X. Marceau F. (2013). Vasopeptidase-activated latent ligands of the histamine receptor-1. *Int. Immunopharmacol.* 17 677–683. 10.1016/j.intimp.2013.08.014 24016859

[B14] Gobeil F. Pheng L. H. Badini I. Nguyen-Le X. K. Pizard A. Rizzi A. (1996). Receptors for kinins in the human isolated umbilical vein. *Br. J. Pharmacol.* 118 289–294. 10.1111/j.1476-5381.1996.tb15401.x 8735629 PMC1909628

[B15] Griol-Charhbili V. Messadi-Laribi E. Bascands J. L. Heudes D. Meneton P. Giudicelli J. F. (2005). Role of tissue kallikrein in the cardioprotective effects of ischemic and pharmacological preconditioning in myocardial ischemia. *FASEB J.* 19 1172–1174. 10.1096/fj.04-3508fje 15860541

[B16] Heitsch H. (2003). The therapeutic potential of bradykinin B_2_ receptor agonists in the treatment of cardiovascular disease. *Expert Opin. Invest. Drugs* 12 759–770. 10.1517/13543784.12.5.759 12720488

[B17] Hock F. J. Wirth K. Albus U. Linz W. Gerhards H. J. Wiemer G. (1991). Hoe 140 a new potent and long acting bradykinin-antagonist: in vitro studies. *Br. J. Pharmacol.* 102 769–773. 10.1111/j.1476-5381.1991.tb12248.x 1364851 PMC1917958

[B18] Ishida H. Scicli A. G. Carretero O. A. (1989). Role of angiotensin converting enzyme and other peptidases in in vivo metabolism of kinins. *Hypertension* 14 322–327. 10.1161/01.HYP.14.3.322 2548961

[B19] Jean M. Gera L. Charest-Morin X. Marceau F. Bachelard H. (2016). In vivo effects of bradykinin B_2_ receptor agonists with varying susceptibility to peptidases. *Front. Pharmacol.* 6:306 10.3389/fphar.2015.00306 PMC4709452 26793104

[B20] Kakoki M. McGarrah R. W. Kim H. S. Smithies O. (2007). Bradykinin B_1_ and B_2_ receptors both have protective roles in renal ischemia/reperfusion injury. *Proc. Natl. Acad. Sci. U.S.A.* 104 7576–7581. 10.1073/pnas.0701617104 17452647 PMC1855073

[B21] Koumbadinga G. A. Bawolak M. T. Marceau E. Adam A. Gera L. Marceau F. (2010). A ligand-based approach to investigate the expression and function of angiotensin converting enzyme in intact human umbilical vein endothelial cells. *Peptides* 31 1546–1554. 10.1016/j.peptides.2010.04.027 20452384

[B22] Leeb-Lundberg L. M. Marceau F. Müller-Esterl W. Pettibone D. J. Zuraw B. L. (2005). International union of pharmacology. XLV. Classification of the kinin receptor family: from molecular mechanisms to pathophysiological consequences. *Pharmacol. Rev.* 57 27–77. 10.1124/pr.57.1.2 15734727

[B23] Marceau F. deBlois D. Petitclerc E. Levesque L. Drapeau G. Audet R. (2010). Vascular smooth muscle contractility assays for inflammatory and immunological mediators. *Int. Immunopharmacol.* 10 1344–1353. 10.1016/j.intimp.2010.08.016 20831918

[B24] Marceau F. Hess J. F. Bachvarov D. R. (1988). The B_1_ receptors for kinins. *Pharmacol. Rev.* 50 357–386. 9755287

[B25] Marceau F. Levesque L. Drapeau G. Rioux F. Salvino J. M. Wolfe H. R. (1994). Effects of peptide and nonpeptide antagonists of bradykinin B_2_ receptors on the venoconstrictor action of bradykinin. *J. Pharmacol. Exp. Ther.* 269 1136–1143. 8014858

[B26] Marceau F. Regoli D. (2004). Bradykinin receptor ligands: therapeutic perspectives. *Nat. Rev. Drug Discov.* 3 845–852. 10.1038/nrd1522 15459675

[B27] Marketou M. Kintsurashvili E. Papanicolaou K. N. Lucero H. A. Gavras I. Gavras H. (2010). Cardioprotective effects of a selective B_2_ receptor agonist of bradykinin post-acute myocardial infarct. *Am. J. Hypertens.* 23 562–568. 10.1038/ajh.2010.20 20186129

[B28] Moreau M. E. Garbacki N. Molinaro G. Brown N. J. Marceau F. Adam A. (2005). The kallikrein–kinin system: current and future pharmacological targets. *J. Pharmacol. Sci.* 99 6–38. 10.1254/jphs.SRJ05001X 16177542

[B29] Morissette G. Couture J. P. Désormeaux A. Adam A. Marceau F. (2008). Lack of direct interaction between enalaprilat and the kinin B_1_ receptors. *Peptides* 29 606–612. 10.1016/j.peptides.2007.12.004 18201802

[B30] Muto Y. Suzuki K. Sato E. Ishii H. (2003). Carboxypeptidase B inhibitors reduce tissue factor-induced renal microthrombi in rats. *Eur. J. Pharmacol.* 461 181–189. 10.1016/S0014-2999(03)01297-4 12586213

[B31] Plummer T. H. Ryan T. J. (1981). A potent mercapto bi-product analogue inhibitor for human carboxypeptidase N. *Biochem. Biophys. Res. Commun.* 98 448–454. 10.1016/0006-291X(81)90860-3 7225104

[B32] Potier L. Waeckel L. Vincent M. P. Chollet C. Gobeil F. Jr. Marre M. (2013). Selective kinin receptor agonists as cardioprotective agents in myocardial ischemia and diabetes. *J. Pharmacol. Exp. Ther.* 346 23–30. 10.1124/jpet.113.203927 23591995

[B33] Pretorius M. Rosenbaum D. Vaughan D. E. Brown N. J. (2003). Angiotensin converting enzyme inhibition increases human vascular-type plasminogen activator release through endogenous bradykinin. *Circulation* 107 579–585. 10.1161/01.CIR.0000046268.59922.A4 12566370

[B34] Rastaldo R. Paolocci N. Chiribiri A. Penna C. Gattullo D. Pagliaro P. (2001). Cytochrome P-450 metabolite of arachidonic acid mediates bradykinin-induced negative inotropic effect. *Am. J. Physiol. Heart Circ. Physiol.* 280 H2823–H2832. 10.1152/ajpheart.2001.280.6.H2823 11356641

[B35] Regoli D. Barabe J. (1980). Pharmacology of bradykinin and related kinins. *Pharmacol. Rev.* 2 1–46. 7015371

[B36] Rhaleb N. E. Rouissi N. Jukic D. Regoli D. Henke S. Breipohl G. (1992). Pharmacological characterization of a new highly potent B_2_ receptor antagonist (Hoe 140: D-Arg-[Hyp3,Thi5,D-Tic7,Oic8]bradykinin). *Eur. J. Pharmacol.* 210 115–120. 10.1016/0014-2999(92)90661-M 1601053

[B37] Sharma J. N. Al-Banoon A. (2012). The role of inflammatory mediator bradykinin in cardiovascular and renal diseases. *Open Access Sci. Rep.* 1:142.

[B38] Veeravalli K. K. Akula A. (2004). Involvement of nitric oxide and prostaglandin pathways in the cardioprotective actions of bradykinin in rats with experimental myocardial infarction. *Pharmacol. Res.* 49 23–29. 10.1016/j.phrs.2003.07.010 14597148

[B39] Wirth K. Hock F. J. Albus U. Linz W. Alpermann H. G. Anagnostopoulos H. (1991). Hoe 140 a new potent and long acting bradykinin-antagonist: in vivo studies. *Br. J. Pharmacol.* 102 774–777. 10.1111/j.1476-5381.1991.tb12249.x 1364852 PMC1917928

